# Mixoplankton and mixotrophy: future research priorities

**DOI:** 10.1093/plankt/fbad020

**Published:** 2023-06-09

**Authors:** Nicole C Millette, Rebecca J Gast, Jessica Y Luo, Holly V Moeller, Karen Stamieszkin, Ken H Andersen, Emily F Brownlee, Natalie R Cohen, Solange Duhamel, Stephanie Dutkiewicz, Patricia M Glibert, Matthew D Johnson, Suzana G Leles, Ashley E Maloney, George B Mcmanus, Nicole Poulton, Sarah D Princiotta, Robert W Sanders, Susanne Wilken

**Affiliations:** Virginia Institute of Marine Science, William & Mary 1370 Greate Rd., Gloucester Point, VA 23062, USA; Woods Hole Oceanographic Institution, 266 Woods Hole Rd, Woods Hole, MA 02543, USA; NOAA Geophysical Fluid Dynamics Laboratory, 201 Forrestal Rd., Princeton, NJ 08540, USA; Department of Ecology, Evolution, and Marine Biology, University of California, Santa Barbara, 1120 Noble Hall, Santa Barbara, CA 93106, USA; Bigelow Laboratory for Ocean Sciences, 60 Bigelow Dr., East Boothbay, ME 04544, USA; Center for Ocean Life, Natl. Inst. of Aquatic Resources, Technical University of Denmark, Kemitorvet, Bygning 202, Kongens Lyngby 2840, Denmark; Department of Biology, St. Mary’s College of Maryland, 18952 E. Fisher Road, St. Mary’s City, MD 20686, USA; Skidaway Institute of Oceanography, University of Georgia, 10 Ocean Science Circle, Savannah, GA 31411, USA; Department of Molecular and Cellular Biology, The University of Arizona, 1007 E Lowell Street, Tucson, AZ 85721, USA; Center for Global Change Science, Massachusetts Institute of Technology, 77 Massachusetts Ave., Cambridge, MA 02874, USA; Horn Point Laboratory, University of Maryland Center for Environmental Science, 2020 Horns Point Rd, Cambridge, MD 21613, USA; Woods Hole Oceanographic Institution, 266 Woods Hole Rd, Woods Hole, MA 02543, USA; Department of Marine and Environmental Biology, University of Southern California, 3616 Trousdale Parkway, Los Angeles, CA 90089, USA; Geosciences Department, Princeton University, Guyot Hall, Princeton, NJ 08544, USA; Department of Marine Sciences, University of Connecticut, 1080 Shennecossett Rd., Groton, CT 06340, USA; Bigelow Laboratory for Ocean Sciences, 60 Bigelow Dr., East Boothbay, ME 04544, USA; Biology Department, Pennsylvania State University, Schuylkill Campus, 200 University Drive, Schuylkill Haven, PA 17972, USA; Department of Biology, Temple University, 1900 N. 12th St., Philadelphia, PA 19122, USA; Department of Freshwater and Marine Ecology, Institute for Biodiversity and Ecosystem Dynamics, University of Amsterdam, Science Park 904, Amsterdam, 1098 XH, The Netherlands

**Keywords:** mixoplankton, mixotrophy, evolution, trade-offs, biogeography, food-webs, methods

## Abstract

Phago-mixotrophy, the combination of photoautotrophy and phagotrophy in mixoplankton, organisms that can combine both trophic strategies, have gained increasing attention over the past decade. It is now recognized that a substantial number of protistan plankton species engage in phago-mixotrophy to obtain nutrients for growth and reproduction under a range of environmental conditions. Unfortunately, our current understanding of mixoplankton in aquatic systems significantly lags behind our understanding of zooplankton and phytoplankton, limiting our ability to fully comprehend the role of mixoplankton (and phago-mixotrophy) in the plankton food web and biogeochemical cycling. Here, we put forward five research directions that we believe will lead to major advancement in the field: (i) evolution: understanding mixotrophy in the context of the evolutionary transition from phagotrophy to photoautotrophy; (ii) traits and trade-offs: identifying the key traits and trade-offs constraining mixotrophic metabolisms; (iii) biogeography: large-scale patterns of mixoplankton distribution; (iv) biogeochemistry and trophic transfer: understanding mixoplankton as conduits of nutrients and energy; and (v) *in situ* methods: improving the identification of *in situ* mixoplankton and their phago-mixotrophic activity.

## INTRODUCTION

Protistan plankton have traditionally been categorized dichotomously as either zooplankton (heterotrophs) or phytoplankton (photoautotrophs). However, substantial evidence in recent decades has shown that many protists fall on a spectrum between these two trophic strategies, engaging in both photoautotrophy and heterotrophy (Faure *et al.*, 2019; Flynn *et al.*, 2013; Leles *et al.*, 2017; Stoecker *et al.*, 2017). These protists have been referred to as mixotrophs or, more recently, mixoplankton. When the standard definition of mixotrophs (a combination of autotrophy and heterotrophy) is applied to plankton, the use of heterotrophy can refer to osmotrophy and phagotrophy. However, it has been argued that since the use of osmotrophy does not differentiate planktonic mixotrophs from prokaryotic and eukaryotic phytoplankton, defining mixotrophic plankton as the use of photoautotrophy and phagotrophy is more useful (Table 1, Flynn *et al.*, 2013). Since the term mixotroph applies to many non-aquatic organisms, here, we will use the term mixoplankton, which emphasizes the use of photoautotrophy and phagotrophy by a plankton (Flynn *et al.*, 2019; Glibert and Mitra, 2022). Due to their dual role as producers and consumers, mixoplankton are expected to be important both to ecological and biogeochemical dynamics, with impacts distinct from their specialized heterotrophic and photoautotrophic competitors (Hartmann *et al.*, 2012; Worden *et al.*, 2015; Duhamel *et al.*, 2019). Their inclusion in food webs represents a paradigm shift in aquatic ecosystem ecology (Glibert and Mitra, 2022). However, research conducted on mixoplankton has been historically limited in scope.

Mixoplankton were previously perceived as peculiar phytoplankton with the unique capability to ingest prey when subjected to growth-limiting conditions. As such, most studies on mixoplankton have focused on the environmental factors that drive an increase in ingestion rates under controlled experimental conditions in the lab. While these studies have proven to be invaluable in helping the identification of mixoplankton species, their categorization into functional groups (Stoecker, 1998; Mitra *et al.*, 2016), and the quantification of their numerical importance in marine (Hartmann *et al.*, 2012; Leles *et al.*, 2017; Hansson *et al.*, 2019; Millette *et al.*, 2021; Glibert and Mitra, 2022) and freshwater ecosystems (Berninger *et al.*, 1992; Bergström *et al.*, 2003; Pålsson and Granéli, 2003; Princiotta and Sanders, 2017), there remains a substantial lack of basic knowledge on the metabolic constraints and ecological impacts of mixoplankton. It is therefore imperative to expand the research being done on mixoplankton to fully grasp their roles in aquatic ecosystems.

Recently, there has been an expansion in the research questions being addressed regarding mixoplankton. Some of the exciting new research includes comparing how copepods are impacted by a diet of mixoplankton vs heterotrophs or photoautotrophs (Traboni *et al.*, 2020), using multidimensional analysis to identify potentially unclassified mixoplankton (Schneider *et al.*, 2020), exploring dilution method modifications to target mixoplankton (Duarte Ferreira *et al.*, 2021) and adapting stable isotope methods to quantify the concentration of nutrients being assimilated into a mixoplankter through both trophic modes (Terrado *et al.*, 2017). These recent projects are examples of ways mixoplankton research has grown, but they represent merely a few of many possibilities in this understudied field. Moving forward, an emphasis on projects that bring scientists together across multiple disciplines would help to diversify the types of questions being addressed about mixoplankton and phago-mixotrophy.

There are numerous avenues that can be explored to expand phago-mixotrophy research. Increasingly, models have been applied to investigate the impacts of phago-mixotrophy in planktonic food webs and biogeochemical cycling (Mitra *et al.*, 2016; Ward and Follows, 2016; Li *et al.*, 2022). These models make important predictions, such as the expected increase in trophic transfer efficiency when mixoplankton are included in the simulations (Ward and Follows, 2016; Leles *et al.*, 2021), that provide hypotheses for empirical testing. As an example of how models can guide experiments, Traboni *et al.* (2021) recently experimentally tested a variety of ways copepods were affected when fed an actively grazing mixoplankter compared to that same prey raised as a strict photoautotroph (prey absent). They found that even though the number of prey cells being ingested did not vary much across treatments, the amount of carbon, nitrogen and phosphorus being ingested was highest when the copepods were fed the phago-mixotrophically active prey. Results from this study add empirical support to the hypothesis that mixoplankton transfer more nutrients to higher trophic levels or that their presence increases trophic transfer efficiency. A diversity of studies, like Traboni *et al.* (2021), are needed to fully address the impact of phago-mixotrophy on trophic transfer and empirically test hypotheses suggested by Ward and Follows (2016) and Leles *et al.* (2021). Additionally, knowledge gaps regarding the constraints on mixoplankton metabolism need to be filled to develop new models and/or better inform current ecosystem models, including their parameterization.

The need for a more complete understanding of phago-mixotrophy’s ecological and biogeochemical roles is pressing considering changing ocean conditions that may favor mixoplankton. Rising sea surface temperatures are increasing water column stratification (Hambright *et al.*, 1994; Sallée *et al.*, 2021); this, in combination with alterations in ocean circulation, are likely to lead to increasing nutrient scarcity in the photic zone over large regions of the ocean. Given that studies have suggested bacterivory rates by smaller phytoplankton (<20 μm) may increase under nutrient limitation (Arenovski *et al.*, 1995; Christaki *et al.*, 1999; Duhamel *et al.*, 2019), nutrient scarcity should favor mixoplankton over strict photoautotrophs (Glibert, 2020; Bock *et al.*, 2021; Courboulès *et al.*, 2021). On the other hand, increased nutrient and organic matter loading from the terrestrial watershed are expected for some inland and coastal waters. This might impact mixoplankton via increased population growth of bacterial prey and limited availability of light in the water column due to absorption by humic substances (Wilken *et al.*, 2017). Furthermore, studies using laboratory cultures have demonstrated that some strains of mixoplankton increase reliance on phagotrophic processes under high-temperature scenarios (Wilken *et al.*, 2013; Lin *et al.*, 2018; Cabrerizo *et al.*, 2019), suggesting that mixoplankton metabolism will be altered under predicted future climate conditions. However, shifts in environmental conditions expected in the future vary between different marine and freshwater habitats. It is still uncertain what types of mixoplankton will become more dominant and how their metabolism will shift in response to complex and interactive climate change stressors (Princiotta *et al.*, 2016; Calbet and Saiz, 2022). This hampers their inclusion in Earth Systems Models that project ecosystem shifts under climate change scenarios.

**Fig. 1:**
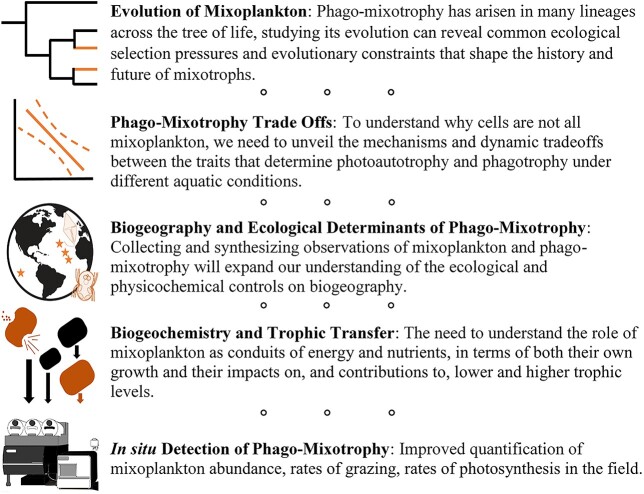
Summary of our outlined mixoplankton and phago-mixotrophy research priorities. Icons by Holly Moeller.

Here, we present the five research priorities that we identified to address the most pressing gaps in mixoplankton research (Fig. 1). The order presented is not indicative of their relative importance but is the result of how each section relates to the others. The *evolution of mixoplankton* focuses on how mixoplankton arise from heterotrophs that incorporate chloroplasts from other organisms (or host photosynthetic endosymbionts). Yet unlike strictly photoautotrophic eukaryotic plankton, mixoplankton have not abandoned phagotrophy, and evolutionary processes will continue to integrate and shape the balance between these two nutritional pathways in the future oceans. This mixed strategy suggests that mixoplankton experience *trade-offs* in investing in both trophic modes and that abiotic and biotic conditions must exist that favor mixoplankton over strict photoautotrophs and heterotrophs. Identifying these trade-offs can help us to identify *biogeographical* regions that hypothetically favor mixoplankton and high mixotrophic activity, which can be tested through empirical studies. Where mixoplankton play a large role in the plankton community, we can then seek to understand their role in *biogeochemical cycling and trophic transfer*. To study any of these research priorities on a large scale, we need to vastly improve or modify *in situ methods*.

While these research priorities are as broad and all-encompassing as possible, not all possible research topics are covered. Some important topics that are briefly touched upon here but not discussed in detail include the response of mixoplankton to climate change, their contribution to nutrient export and the role of phago-mixotrophy in some harmful algae bloom species. Our selected priorities align with common research themes for phytoplankton and zooplankton and highlight the untapped potential of mixoplankton research beyond the limited scope of most studies to date. The capacity of mixoplankton to combine two major trophic modes means that the methods and theory historically developed to apply to either phytoplankton or zooplankton obscures their critical functional role (Millette *et al.*, 2018). Addressing the five priorities we outline here will bring our knowledge of mixoplankton and phago-mixotrophy into alignment with the importance of these organisms to aquatic ecosystems. Furthermore, the research priorities highlighted here may not only help to improve our understanding of mixoplankton and phago-mixotrophy but also inspire new approaches to advance these priorities.

### Evolution

**Fig. 2:**
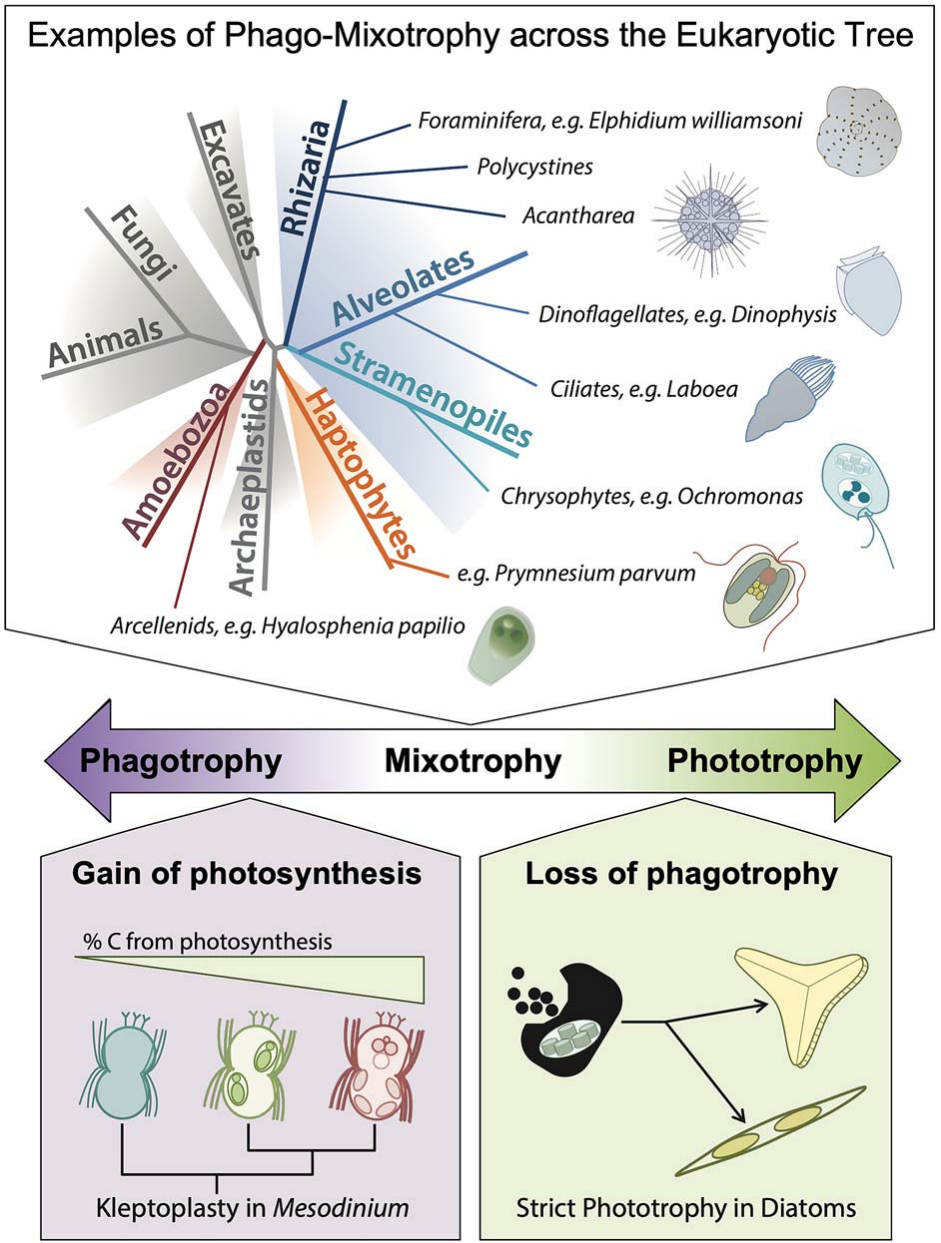
Mixoplankton span an evolutionary and physiological spectrum. Top: Phago-mixotrophy is found across numerous lineages of the eukaryotic tree of life (some examples shown). Middle: These mixoplankton span a gradient of reliance on photosynthesis, from almost completely phagotrophic to almost completely photoautotrophic. Bottom: As such, mixoplankton also present an opportunity to study the evolutionary transition from phagotrophy to phototrophy, particularly by examining two key evolutionary transitions: (i) the evolutionary gain of phototrophy (here, exemplified by kleptoplasty in the *Mesodinium* genus, in which lineages become increasingly photosynthetic as they become more evolutionarily derived; Johnson *et al.*, 2016) and (ii) the evolutionary loss of phagotrophy (here, exemplified by the diatoms, which lost phagotrophy prior to undergoing an extensive radiation). Illustration by Catherine S. Raphael (NOAA/GFDL) and Holly Moeller.

Phago-mixotrophic lineages are widespread across the tree of life (Fig. 2), representing many different origins of and strategies for combining photosynthesis and phagotrophy within the same cell (Stoecker *et al.*, 2017). Research efforts focused on synthesizing physiological and ecological data from diverse mixoplankton can be used to identify the ecological and evolutionary pressures that govern the gain and loss of photosynthesis and phagotrophy. Furthermore, understanding the evolutionary history of mixoplankton in response to major environmental shifts will enhance predictions of their adaptability to future aquatic ecosystems.

The two fundamental energy-acquiring metabolisms of mixoplankton, photoautotrophy and phagotrophy, evolved before they were combined within the first photosynthetic eukaryote; phagocytosis is an ancient eukaryotic trait and oxygenic photosynthesis originated with cyanobacteria. Genotypically heterotrophic eukaryotes acquired photosynthetic capability via the domestication, incorporation and spread of cyanobacterial endosymbionts (Delwiche, 1999). This process of plastid (i.e. chloroplast) acquisition likely resulted in the first mixoplankter as a starting point for evolution of eukaryotic phytoplankton. Today, some capacity for phagotrophy has been retained in all major eukaryotic phytoplankton lineages except for diatoms (Raven, 2013). A complete loss of phagotrophy and evolution toward a purely photosynthetic lifestyle appears to be the exception rather than the norm among protists.

We posit that the diversity of mixoplanktonic lineages can shed light on the evolutionary transition from phagotrophy to photoautotrophy. Although eukaryotic chloroplasts appear to share a common ancestor, reflecting a single primary domestication event (the cercozoan *Paulinella* may represent a second primary event; Marin *et al.*, 2005), at least a half-dozen secondary and tertiary acquisitions of plastids have occurred (Delwiche, 1999; Falkowski *et al.*, 2004; Keeling, 2010). While the precise number of independent plastid acquisitions within the eukaryotic tree of life is debated, lineages from vastly different genomic backgrounds have transitioned from phagotrophy to phago-mixotrophy. Through a comparative study of these diverse lineages, we can identify commonalities in ecological selection pressures, metabolic and cellular gene pathways and physiological capacity that allow for the evolution of phago-mixotrophy and select for its maintenance today. Non-constitutive mixoplankton (NCM) could exemplify hypothetical transition pathways from phagotrophy to phago-mixotrophy. In some taxa, organelle sequestration can support most metabolic demands of the host and, when the necessary genetic material is present in either a stolen nucleus or the host’s genome, allow for the control of sequestered chloroplasts (Johnson *et al.*, 2007; Onuma and Horiguchi, 2015). This could facilitate adaptation to obligate photosynthesis while perhaps setting the table for the potential of gene transfer. It has been suggested that these types of NCM organisms may represent stages on the road to constitutive mixoplankton (CM) (i.e. evolutionary incorporation of their own chloroplasts; Brown *et al*., n.d.; Gast *et al.*, 2007; Mansour and Anestis, 2021). However, we do not know the extent to which organisms such as the ciliate *Mesodinium rubrum* (organelle-theft, Hansen *et al.*, 2013), the dinoflagellate *Kryptoperidinium foliaceum* (diatom endosymbiont, Figueroa *et al.*, 2009) and the cercozoan *Paulinella* (with recently-incorporated cyanobacterium, Nakayama and Ishida, 2009) represent stages in evolution of phototrophy or idiosyncratic adaptations. These unique organisms have thus far provided insights into the ecology of phago-mixotrophy.

While much has been learned about the process of plastid acquisition, less is known about the consequences of retaining phagotrophy across evolutionarily diverse mixoplankton. The capacity for phagotrophy is retained in most photosynthetic eukaryotic lineages. However, some lineages can sustain growth in darkness via phagotrophy while others require light to maintain any positive growth via photosynthesis (Stoecker *et al.*, 2017). The reasons underlying how a mixoplankter arrived at its current position on the continuum between phagotrophic and photoautotrophic traits often remain unknown, and additional dimensions might be needed to address this research gap. For example, reliance on photosynthesis in two mixotrophic haptophytes appears to be associated with flagellar arrangement and beating patterns that provide insufficient prey encounters to fully sustain energetic requirements on phagotrophy alone (Dölger *et al.*, 2017). However, the root cause of this trade-off is unknown: are these haptophytes reliant on photosynthesis because they are poor predators, or are they poor predators because they put so much effort into photosynthesis? Understanding the constraints acting on the development of both trophic modes within each host and endosymbiont lineage, each with their inherent characteristics, will be required to resolve the microevolutionary processes that continue to shape mixoplanktonic physiology and ecology today.

We suggest that more research be directed at identifying the ecological regimes that select for phago-mixotrophy, or conversely its loss during specialization toward pure photoautotrophy or phagotrophy. To date, some eco-evolutionary modeling studies have explored the environmental conditions under which mixoplankton may evolve toward specialization (Troost *et al.*, 2005a, b) and quantified the relationship between mixoplankton traits and competitive coexistence with photoautrotrophs and heterotrophs (Crane and Grover, 2010; Berge *et al.*, 2017). Yet a few empirical tests of these ideas exist, in part because of the near impossibility of observing mixoplankton evolution in real time. However, at least two approaches exist to indirectly address these challenges.

First, improved databases of mixoplankton biogeography will allow us to link mixoplankton type and abundance with ecological drivers (see section Biogeography and Ecological Determinants). Using these data, we can identify the ecological settings that select for phago-mixotrophy and validate eco-evolutionary models. Second, we can perform comparative studies in the laboratory using closely related species that differ in their degree of photosynthesis versus phagotrophy. For example, kleptoplastidic (plastid-stealing) NCM ciliates from the genus *Mesodinium* span a gradient of reliance on photosynthesis (Hansen *et al.*, 2013; Moeller and Johnson, 2018; Edwards *et al.*, 2023; Fig. 2) yet share a genetic background, so laboratory competition studies can quantify niche partitioning among these lineages to reconstruct past selection pressures. Additionally, CMs vary in their metabolic plasticity from obligate to facultative phago-mixotrophy (e.g. *Ochromonas*; Lie *et al.*, 2018; Moeller *et al.*, 2019; Wilken *et al.*, 2020) and can be used to understand the fine-scale evolution of metabolic strategies. Using CMs, we can quantify trade-offs between photosynthesis and phagotrophy (see section Traits and Trade-offs), measure phenotypic plasticity within mixoplankton lineages and ask how this phenotypic plasticity evolves. In both cases, comparative genomics between mixoplankton with different nutritional strategies, or with closely related heterotrophic or photoautotrophic ancestors, will help to illuminate gene acquisition, loss and metabolic integration that helps to facilitate phago-mixotrophy.

We suggest that, by understanding the evolutionary origins of mixoplankton, we may be better able to predict their evolutionary futures. In areas of the ocean that are stratifying due to warming (Polovina *et al.*, 2008; Sallée *et al.*, 2021), mixoplankton are expected to become increasingly dominant because phagotrophic modes of nutrition will allow them to continue to obtain nutrients in oligotrophic waters (Mitra *et al.*, 2014). Yet, as unicellular organisms with short generation times and large population sizes (Rengefors *et al.*, 2017), mixoplankton may also rapidly evolve in response to new environmental regimes. Such rapid evolution has been observed in phytoplankton, which can reduce respiratory costs (Barton *et al.*, 2020), increase thermal tolerances (Padfield *et al.*, 2016; O’Donnell *et al.*, 2018) and tolerate reduced pH (Collins *et al.*, 2014). However, phytoplankton adaptive evolution capability can be inhibited when nutrients are limiting (Aranguren-Gassis *et al.*, 2019). Based on the thermal scaling of photosynthesis and aerobic respiration (Rose and Caron, 2007), mixoplankton are predicted to become more phagotrophic at warmer temperatures (Wilken *et al.*, 2013). This has been confirmed in some studies (Wilken *et al.*, 2013; Cabrerizo *et al.*, 2019) but not others (Princiotta *et al.*, 2016). Both evolution experiments (Lepori-Bui *et al.*, 2022) and eco-evolutionary modeling (Gonzalez *et al.*, 2022) suggest that mixoplankton evolution will amplify this trend toward increased phagotrophy. We propose that future experimental evolution studies should encompass mixoplankton that spans a gradient of reliance on photoautotrophy and phagotrophy and have varied capacity for phenotypic plasticity. Such studies will simultaneously enable better predictions of the adaptive responses of the extant diversity of mixoplankton and provide insight into the causes and consequences of metabolic specialization.

### Traits and trade-offs

Understanding the relative cellular gains and costs for engaging in phago-mixotrophy is one approach to understand when and why mixoplankton may dominate in the plankton community (Dolan and Pérez, 2000). Typically, this question is phrased as follows: under what conditions will mixoplankton outcompete strict photoautotrophs and heterotrophs? Here, we propose that asking an alternate question will help us gain new insights: why are not all protists mixoplankton? The dynamic trade-offs between the organismal properties that determine rates of photosynthesis and prey ingestion within mixoplankton can be better revealed when contrasted against strictly photoautotrophic (e.g. diatoms and cyanobacteria) or heterotrophic (e.g. *Protoperidinium* spp. and *Paraphysomonas* spp.) plankton. From an evolutionary perspective, if ancestors of protistan phototrophs were able to engage in kleptoplasty (Hehenberger *et al.*, 2019; see section Evolution), then why have some of these functionalities been lost as a trade-off for pure phototrophy? A major challenge in phago-mixotrophy research is the identification of which traits and associated trade-offs are key to representing mixoplankton metabolisms in aquatic ecosystems. A quantitative understanding of the metabolic trade-offs of mixoplankton will allow us to understand why different mixoplankton strategies (=modes) are maintained under certain environmental conditions. Eventually, this will allow scientists to develop unifying frameworks for modeling mixoplankton functional groups.

An obvious choice for traits to examine are those related to investments in phototrophy versus phagotrophy (see Fig. 1 in Andersen *et al.*, 2015). In most cases, mixoplankton growth is a result of synergies between these two trophic strategies; mixoplankton will increase the use of their alternative nutrient strategy (ingestion for CMs and photosynthesis for NCMs) when conditions become unsuitable for their preferred nutrient strategy. While phago-mixotrophy provides greater flexibility in terms of resource acquisition strategies, it also constrains growth by reducing grazing rates and incurring additional metabolic costs (Raven, 1997; Stoecker, 1998). A lower average growth rate can be a consequence of allocating resources to the synthesis and maintenance of both phototrophic and phagotrophic structures (Raven, 1997; Ward *et al.*, 2011). Furthermore, space limitations within the cell or on the cell membrane can constrain investments in both acquisition strategies (Raven, 1997; Dolan and Pérez, 2000; Ward *et al.*, 2011; Chakraborty *et al.*, 2017; Serra-Pompei *et al.*, 2019). Based on these costs, pioneering theoretical studies proposed that mixoplankton are favored under nutrient or light limitation (Thingstad *et al.*, 1996; Stoecker, 1998; Stickney *et al.*, 2000), especially when the prey have a nutritional composition complementary to that of their predators see section Biogeochemistry and Trophic Transfer) (e.g. Glibert and Burkholder, 2011; Lundgren *et al.*, 2016; Lin *et al.*, 2017).

Field and laboratory observations have corroborated that mixoplankton species typically respond to changes in one primary growth limiting factor that triggers an increase in utilization of their alternate nutrient mode (Zubkov and Tarran, 2008; McKie-Krisberg *et al.*, 2015; Millette *et al.*, 2017). However, current methodological limitations mean that direct measurements of the balance between phototrophy and phagotrophy in any mixoplankton under different conditions is difficult. Previous theoretical mechanistic models based on key resource-harvesting traits (i.e. photosynthesis, inorganic nutrient uptake and phagotrophy) have found that cellular investments in photosynthesis and inorganic nutrient uptake by mixoplankton were higher during a typical subarctic/temperate spring when light and nutrients are replete, while phagotrophy was preferentially invested in during summer conditions typified by low inorganic nutrient concentrations (Berge *et al.*, 2017; Chakraborty *et al.*, 2017). We suggest that future research directly measure nutrients acquired from both trophic strategies and understand what causes variability in the balance between both strategies. These empirical data are needed to understand the optimal combination of phototrophy and phagotrophy in any mixoplankton that will allow for continued survival and maximal growth under a given set of environmental conditions.

Most mechanistic models and laboratory experimentation to date have focused on CMs (McKie-Krisberg *et al.*, 2015; Berge *et al.*, 2017; Edwards, 2019; Li *et al.*, 2022), but the traits and trade-offs governing NCM metabolism are less studied. While NCMs also occur on a continuum of strategies, the cellular constraints are likely to be different, as they do not contain constitutive chloroplasts and rely on prey ingestion as their primary energy source. Therefore, the degree to which these mixoplankton control their acquired phototrophic machinery (e.g. whether they can replicate it, how long they can keep plastids functional and their dependence on specific or diverse prey taxa for photosynthetic machinery) are expected to influence their metabolisms (Hansen, 2011; Johnson, 2011; Mitra *et al.*, 2016). For example, McManus *et al.* (2012) showed that the ciliate *Strombidium rassoulzadegani* grew more slowly in the light when fed a prey whose chloroplasts it could not retain (dinoflagellate), compared to a prey whose chloroplasts remained functional in the ciliates (chlorophyte or cryptophyte). However, *S. rassoulzadegani* grew better in the dark when feeding on the dinoflagellate, suggesting that the retained chloroplasts from the other algae imposed a cost to the ciliate. In the ciliate *M. rubrum*, prey organelles can only be exploited from the *Teleaulax/Plagioselmis/Geminigera* lineage of cryptophytes, even though other cryptophyte species are sometimes ingested (Park *et al.*, 2007; Hansen *et al.*, 2012; Peltomaa and Johnson, 2017). Commensurate with the high specificity with which *M. rubrum* selects and uses prey organelles are adaptations that make them more reliant upon photosynthesis. This includes the retention of a transcriptionally active prey nucleus, the division of plastids and mitochondria and the ability to fully exploit their prey’s metabolism and biosynthetic pathways (Johnson *et al.*, 2007; Lasek-Nesselquist *et al.*, 2015; Altenburger *et al.*, 2021). Such trade-offs between prey specificity and reliance upon photosynthesis appear to be a common theme among NCMs. A focus on quantifying the metabolic costs associated with NCMs is key for understanding when and why NCMs engage in phago-mixotrophy.

Another key functional trait potentially impacting mixoplankton’s metabolism is cell size (Chisholm, 1992; Finkel *et al.*, 2010). Recent modeling studies that resolve the plankton community based on allometric relationships have posited a relationship between organism size and mixoplankton metabolism (Ward and Follows, 2016; Chakraborty *et al.*, 2017; Chakraborty *et al.*, 2020). However, beyond the broad size ranges compiled in Andersen *et al.* (2015), systematic, empirical evidence for such a relationship has yet to be established. These modeling results suggest that (i) smaller organisms (picoplankton and small nanoplankton) are predominantly phototrophic because they get high fluxes of light and dissolved inorganic nutrients; (ii) larger organisms (large microplankton and mesozooplankton) are predominantly phagotrophic because the relative gain of nutrients from phagotrophy is much greater than diffusive nutrient uptake; (iii) intermediate-sized organisms (large nanoplankton and small microplankton) span the entire spectrum of phago-mixotrophy, as phototrophic affinity scales with surface area and phagotrophic affinity scales with cell volume; and (iv) the range of mixoplankton sizes is largest in oligotrophic environments (high light; low nutrients) because the relative advantage of phago-mixotrophy is highest under nutrient stress. However, since these results are primarily from theoretical modeling studies, future empirical field and laboratory studies are needed to establish whether mixoplankton have a functional relationship with their cell size.

Beyond investments in phototrophy versus phagotrophy and size, other traits such as toxin production, defense strategies, energy storage (e.g. lipid metabolism) and the scavenging of reactive oxygen species are also expected to be important to understand mixoplankton trade-offs. However, testing the importance of different traits, one-by-one, will be time consuming and involve a large amount of effort. Multidimensional trait-based approaches can be an effective way to identify additional key traits and constraints associated with mixoplankton metabolisms that have been overlooked. High-throughput methods that allow a fast processing of trait data across a range of environmental conditions combined with multivariate analyses can provide new insights into single traits, or combinations of traits, of interest (Argyle *et al.*, 2021; Martini *et al.*, 2021). It has been shown that a small subset of traits can allow the reconstruction of the original complex trait-scape, revealing trade-offs that might not have been apparent by looking only at individual pairs of traits separately (Argyle *et al.*, 2021). While we can start studying the trade-offs of certain individual traits immediately, multidimensional trait-based approaches are a way to start identifying novel traits of particular importance to mixoplankton.

### Biogeography and ecological determinants

**Fig. 3:**
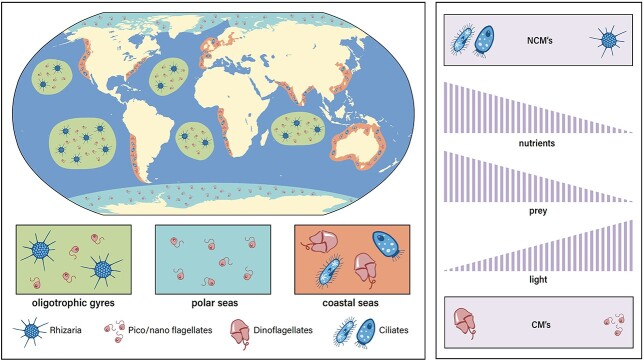
Left: Regions highlighted represent where phago-mixotrophy is predicted to be advantageous over photoautotrophy and heterotrophy, based on historical observations and a trait-based model (Leles *et al.*, 2017; Edwards, 2019; Faure *et al.*, 2019). Within oligotrophic gyres, it would be expected that eSNCMs such as radiolarians and acantharians and small flagellates will dominate the mixoplankton community. Small flagellates are expected to dominate the mixoplankton community in polar seas and large dinoflagellates and ciliates likely dominate in coastal seas. Right: NCMs that acquire chloroplasts via kleptoplasty (gNCM and pSNCM) are likely more prevalent in eutrophic systems, while eSNCMs are likely more prevalent in oligotrophic systems. Small CMs will likely dominate under low-nutrient and prey conditions while large CMS will dominate when light is limiting but nutrients and prey are sufficient. Illustration by Lee Ann Deleo (Skidaway) and Suzana Leles.

The knowledge of the spatial and temporal distribution of mixoplankton (Fig. 3) is crucial to identify regions where they will be a major component of the plankton community, helping us understand conditions that will favor mixoplankton and predicting how the role of mixoplankton and phago-mixotrophy will be altered in a future warmer ocean. To date, few large-scale, comprehensive biogeographical surveys of mixoplankton have been attempted even though such exercises are common for other functional groups (e.g. diatoms, picophytoplankton and diazotrophs) [Marine Ecosystem Biomass Data (MAREDAT); Buitenhuis *et al.*, 2013; Sunagawa *et al.*, 2015]. The assessment of abundance, distribution and phago-mixotrophy on a global scale has been attempted with cell identifications via microscopy (Barton *et al.*, 2013; Leles *et al.*, 2017, 2019), sequence (‘omic)-based efforts (Faure *et al.*, 2019) and modeling (Edwards, 2019). We propose that a high priority for future studies should be to obtain a global perspective of mixoplankton biogeography, including the relative importance of phototrophy vs. phagotrophy in different mixoplankton and under different environmental conditions (see section Traits and Trade-offs). This information will provide a foundation for developing an understanding of the conditions that favor mixoplankton over strict phototrophs and phagotrophs and will be essential for the improvement of numerical and theoretical mechanistic models.

Targeted compilations using historical data have been performed for mixoplankton in the North Atlantic using the Continuous Plankton Recorder (CPR) observations (Barton *et al.*, 2013) and globally with the Ocean Biogeographic Information System database (Leles *et al.*, 2017, 2019). In these studies, species were classified as mixoplankton based on previous experimental evidence for phago-mixotrophy. These historical records demonstrate that mixoplankton are ubiquitous, but their abundance and distribution will vary according to oceanic biomes and functional types. For example, kleptoplastidic NCMs are more abundant in coastal/productive systems, while NCMs harboring symbionts dominate within the oligotrophic ocean gyres (Leles *et al.*, 2017). However, thorough analyses are limited by historical datasets that are spatially and temporally sparse and have been biased toward larger size classes of mixoplankton (Leles *et al.*, 2019). In many cases, we do not know with certainty which taxa are mixoplankton, and these datasets do not address the degree to which they are engaging in each nutritional mode. Nonetheless, these historic records contain a wealth of data that can be used to expand our understanding of mixoplankton biogeographical distribution. We recommend that emphasis should be placed on the evaluation of the relative proportion of phago-mixotrophy compared to strict photoautotrophy and heterotrophy, as opposed to mixoplankton abundance alone, to better understand ecological context and biogeochemical ramifications (see section Biogeochemistry and Trophic Transfer). A community effort is needed to further mine these datasets and develop new ways to constrain taxa being identified as mixoplankton.

Sequencing approaches have also been used to assess both the biogeography of mixoplankton and the functional profiles of phago-mixotrophy. Using amplicon sequencing (metabarcoding), mixoplankton functional types have been classified according to lineage following the approach of previous historical data analyses. This approach has revealed contrasting biogeography similar to that of historical datasets as a result of differing trophic strategies and physiology (Leles *et al.*, 2017; Faure *et al.*, 2019), but has the added potential for identifying mixoplankton across the entire size spectrum and with great taxonomic resolution (Faure *et al.*, 2019). Certain CMs dominate in both eutrophic and oligotrophic environments, while key members of the GNCMs and pSNCMs (Table I) are observed to be relatively abundant in coastal and eutrophic waters and eSNCMs (Table I) potentially dominating in different biomes according to their ability to form colonies or engage in symbiosis (Faure *et al.*, 2019). While metabarcoding studies provide information on lineage-specific biogeographical patterns of mixoplankton, (meta)transcriptomics and (meta)genomics have offered insight into metabolic processes involved in phago-mixotrophy and potential changes in trophic behavior (Alexander *et al.*, 2022; Cohen *et al.*, 2021; Lambert *et al.*, 2022). For example, Lambert *et al.* (2022) classified whole-transcriptome signatures consistent with mixoplankton metabolism in pure cultures and applied this model to field metatranscriptomics to evaluate shifts in trophic strategies across a nutrient gradient in the North Pacific Ocean. Protists predominantly engaged in phagotrophy in low-nitrate subtropical waters and shifted toward phago-mixotrophy and phototrophy in nitrate-rich waters at higher latitudes (Lambert *et al.*, 2022). It remains unclear how similar the metabolic profiles of phagotrophy are among and across diverse mixoplankton lineages and how well ‘omics-based models can discern phago-mixotrophy among natural community members across the global ocean. Though potentially powerful, these biogeographical ‘omic data remain sparse, and measurements of relative gene abundance are not easily translated into biogeochemically meaningful estimates (e.g. ingestion rates and cell/biomass abundance). Large scale field surveys accompanied by laboratory studies assigning taxonomic origin, functional potential and grazing rates are needed to increase confidence in the spatial and temporal resolution of mixoplankton based on molecular signatures. Additionally, new insight into transforming ‘omics data into units that can be compared against model output, are needed.

**Table I:** Definitions of key terms in manuscript. An alphabetical list of definitions for important terms that appear throughout the paper. Terms that end in -troph refer to a type of organism and terms that end in -trophy refer to a specific action taken by an organism. We define both mixoplankton and phago-mixotrophy because they are central to the paper, but for other terms we define the suffix that is most commonly used in the paper. However, both suffixes may be used for any appropriate term. Definitions for CM, eSNCM, GNCM, NCM, and pSNCM come from Mitra *et al.*, (2016) and the definition for mixoplankton comes from Flynn *et al.*, (2019)

Term	Definition
Autotroph	An organism capable of generating its own food
Auxotroph	An organism that cannot synthesize a biomolecule essential for their growth, i.e. vitamins
Constitutive mixoplankton (CM)	Mixoplankton that inherently have chloroplasts
Endosymbiotic specialist NCM (eSNCM)	Mixoplankton that harbor endosymbionts necessary for growth
Generalist NCM (GNCM)	Mixoplankton that can utilize chloroplasts acquired from a range of prey via kleptoplasty
Heterotroph	An organism that obtains nutrients from an outside, organic source for growth and reproduction
Kleptoplasty	The transient retention of functional prey plastids by a mixotrophic consumer
Mixoplankton	A protistan plankton that utilizes photoautotrophy and phagotrophy for growth
Osmotrophy	The act of taking up dissolved organic compounds via osmosis to acquire energy for growth
Phago-mixotrophy	The act of simultaneously utilizing photoautotrophy and phagotrophy for growth as opposed to osmo-mixotrophy, which is the act of simultaneously utilizing photoautotrophy and osmotrophy for growth
Non-constitutive mixoplankton (NCM)	Mixoplankton that lack their own chloroplasts
Phagotrophy	The act of ingesting prey via phagocytosis to acquire energy for growth
Photoautotrophy	The act of utilizing light to generate energy for growth, typically via photosynthesis
Plastidic specialist NCM (pSNCM)	Mixoplankton that can utilize chloroplasts acquired from select prey via kleptoplasty

Finally, mathematical models have been used effectively to elucidate phago-mixotrophy biogeography. Several idealized ecological models have investigated resource competition between mixoplankton and their strict photoautotroph and heterotroph counterparts (Mitra *et al.*, 2014; Edwards, 2019; Stickney *et al.*, 2000). As mentioned in the *Traits and Trade-offs* section, when external nutrients are in low supply, CMs have been hypothesized to dominate because they can acquire nutrients from bacterial or other prey (Mitra *et al.*, 2014). Trait-based models supported by observational data suggest that phago-mixotrophy can also be advantageous at lower latitudes and in nutrient-rich coastal systems when light is limiting due to the acquisition of carbon from prey (Edwards, 2019; Ward, 2019). Another global trait-based ecosystem model predicts that small mixoplankton dinoflagellates are successful in regions where both nutrients and prey are available, i.e. at subpolar and equatorial latitudes (Dutkiewicz *et al.*, 2021). Fewer modeling studies have investigated the ecological niche of NCMs as well as phago-mixotrophy dynamics over depth and seasons (Leles *et al.*, 2021; Schneider *et al.*, 2021). A regional modeling study found phago-mixotrophy to be advantageous among all mixoplankton groups during summer, but differences were observed in other seasons, with small CMs dominating during late winter and NCMs being favored just before the spring bloom (Leles *et al.*, 2021). Although these models represent a significant gain in our understanding of mixoplankton biogeography and some of the controlling mechanisms, we still lack a robust knowledge of mixoplankton trade-offs (see section Traits and Trade-offs) and observational data on mixoplankton and phago-mixotrophy to validate model output from many regions of the ocean.

To uncover the large-scale biogeography of mixoplankton (Fig. 2), significant effort is needed to synthesize and distill data from different and complementary sources, including laboratory studies, field cell densities, pigments, optic measurements, ‘omics approaches and computer models. For example, a recent published dataset brings together taxonomic and genetic data on mixoplankton species as well as information for different functional traits, including cell size, which can be used to guide biogeographical analyses using morphological or meta-omics observations (Mitra *et al.*, 2023). Understanding the mechanisms controlling mixoplankton abundance and activity will allow us to appreciate responses during shifts in determinants, such as a function of eddies or storm events, and the associated biogeochemical ramifications. Furthermore, the knowledge of the ecological roles of different mixoplankton types will allow a better sense of their community function, with the potential to inform models as to the necessary degree of diversity to effectively predict their biogeochemical contributions. Increasing sampling resolution and grazing measurements across different aquatic biomes will be essential for achieving these goals.

### Biogeochemistry and trophic transfer

Quantifying the transformation and transfer of carbon and other nutrients by mixoplankton is necessary to understand the role of phago-mixotrophy in aquatic food webs, improve model predictions of phago-mixotrophy trophic impacts (Flynn and Mitra, 2009) and ultimately characterize the role of mixoplankton in nutrient cycling via remineralization and export (Ward and Follows, 2016; Larsson *et al.*, 2022). The continuum between photoautotrophy and phagotrophy (Flynn *et al.*, 2013; Jeong *et al.*, 2021) within mixoplankton means that they are simultaneously entry points of primary production through photosynthesis and secondary production via ingestion. However, mixoplankton reliance on these trophic modes is highly variable between taxa and environmental conditions (see section Traits and Trade-offs). This variability makes it difficult to constrain general values of carbon (or other nutrient) uptake by mixoplankton via grazing and primary production and the transfer of nutrients through this part of the food web. While the contribution of phago-mixotrophy to the export of carbon from surface to depth is an important but understudied aspect of aquatic nutrient cycling, here, we focus on nutrient allocations both within mixoplankton and as prey sources themselves to further understand the broader biogeochemical consequences of phago-mixotrophy.

To date, the majority of experimental research on mixoplankton trophic activity is culture-based and has focused on how environmental factors (e.g. light and nutrient availability) cause individual mixoplankton species to alter their ingestion of prey (e.g. Li, 2000; McKie-Krisberg *et al.*, 2015; Millette *et al.*, 2017). Generally, these experiments assume that changes in ingestion rates can be used to estimate the assimilation of carbon and nutrients from phagotrophy, with a concomitant decrease in phototrophy. A few culture studies have directly assessed the assimilation efficiency of carbon (and other nutrients) from prey versus photosynthesis by different mixoplankton taxa. Adolf *et al.* (2006) showed that phago-mixotrophically active *Karlodinium micrum* can obtain 32–76% of its carbon from ingestion of cryptophyte prey and 27–69% carbon from photosynthesis. For taxa that are primarily phototrophic (*Dinobryon*), carbon assimilation efficiency from prey ranges from 25 to 54% (Bird and Kalff, 1989; Caron, 1993). CM taxa that rely more on phagotrophy (*Ochromonas* and *Poterioochromonas*) can acquire 43–99% of carbon (and nitrogen) from prey (Caron *et al.*, 1990; Terrado *et al.*, 2017). As recommended in the *Traits and Trade-offs* section, future research that measures nutrient assimilation from both trophic modes, like these limited studies, is needed to properly understand and constrain how different mixoplankton utilize each trophic mode.

It is important to track how nutrients acquired from each trophic strategy are allocated because this has major implications for biogeochemical cycling and trophic transfer efficiency. For example, the internal cycling of metabolites related to the digestion of prey can influence ammonium remineralization (Mitra and Flynn, 2006; Glibert *et al.*, 2016; Glibert and Mitra, 2022). Similarly, it is uncertain how using the alternative nutrient source influences carbohydrate and lipid metabolism of mixoplankton and if it fuels respiratory pathways. If a CM must graze to meet requirements for maintenance metabolism (e.g. maintaining the protein and RNA components of the cell or redox regulation), then resources acquired via phagotrophy might not be transferred to higher trophic levels. However, if a CM is instead grazing to fulfill substantial growth requirements and energy supply, then resources acquired via phagotrophy may be transferred to higher trophic levels. Therefore, zooplankton may benefit from trophic upgrading (the improvement of food quality when mixoplankton ingest and retain limited elements), with implications for food web structure and functioning (Polimene *et al.*, 2016; Glibert and Mitra, 2022). This nutrient acquisition spectrum will be an important next step for mixoplankton modeling efforts and classification. We propose that future studies revisit the balance of nutrient acquisition and allocation by a wide range of mixoplankton taxa under different seasonal and biome conditions, as well as prey quantities and qualities, to better understand whether these nuances are important to trophic transfer by mixoplankton.

Another important aspect of biogeochemical cycling and trophic transfer is the quality of mixoplankton as prey compared to strict photoautotrophs and heterotrophs. The nutritional value of prey to zooplankton consumers is often species-specific and may depend on the relative balance between nutritional modes in the mixoplankton (Weithoff and Wacker, 2007). The nutritional quality of mixoplankton has only begun to be considered, which has restricted our ability to assess the effectiveness of a mixoplankton-enabled trophic link (Flynn and Mitra, 2009). Reports have hypothesized that mixoplankton are better food compared to strict phototrophs under environmental conditions that limit photosynthesis because they are able to maintain their cellular C:N:P ratios and fatty acid composition and concentrations (Katechakis *et al.*, 2005; Moorthi *et al.*, 2017; Fischer *et al.*, 2022). Stability in prey cellular stoichiometry could enhance efficient transfer of carbon to the next trophic level because high variability in elemental ratios increases the mismatch between nutrient demand of predators and that provided by their prey (Moorthi *et al.*, 2017). When prey food quality is poor (i.e. mismatch of the preferred prey C:N:P ratio), copepod gross growth efficiency is lower (Mitra and Flynn, 2005; Bi and Sommer, 2020). Alternatively, toxicity has been documented in several mixoplankton flagellates (Boenigk and Stadler, 2004; Adolf *et al.*, 2006; Hiltunen *et al.*, 2012), which has implications for zooplankton prey quality, especially at times when mixoplankton are abundant.

The consequences of mixoplankton compared to purely photosynthetic phytoplankton as a food source for higher trophic levels remain up to debate (Ptacnik *et al.*, 2004; Vad *et al.*, 2020, 2021). Some previous feeding experiments have included *potentially* mixoplanktonic, non-diatom prey (dinoflagellates and cryptophytes), but only a few studies have explicitly examined the role of phagotrophically active mixoplankton (Ptacnik *et al.*, 2004; Traboni *et al.*, 2021). These studies indicated that phago-mixotrophically active prey did support or improve copepod egg production relative to photoautotrophic prey under nutrient-limiting conditions, but they are just the beginning of the research needed. We suggest future laboratory and field studies concurrently examine the nutrient stoichiometry and fatty acid composition of mixoplankton prey and food preferences of different zooplankton taxa. This would entail ingestion experiments in the laboratory and field, as well as field assessments of zooplankton grazing (gut content analysis). Both laboratory and field research should assess not only the effect on zooplankton growth but include egg production and viability. It is important to examine a wide range of zooplankton taxa because they have different growth and reproductive strategies/timing (e.g. some have lipid stores that help them to over-winter and reproduce in early spring, while others need to acquire sufficient resources to reproduce; Lee *et al.*, 2006; Cavallo and Peck, 2020). To integrate mixoplankton nutrition into our understanding of ecosystem function, we must first elucidate their role as a dietary resource.

The role of mixoplankton in plankton communities and ecosystems depends on the balance between different sources of carbon and energy (phototrophy and phagotrophy), as well as sources of major nutrients and essential elements (dissolved and particulate). The balance likely affects the biochemical transformations of carbon and nutrients and consequently the food quality of mixoplankton for higher trophic levels. New approaches (see section *In situ* Methods Development) will be needed to provide a longer-term quantification of the links between mixoplankton protists and other components of planktonic food webs, as well as investigating their contribution to particulate carbon flux.

### *In situ* methods development

**Fig. 4:**
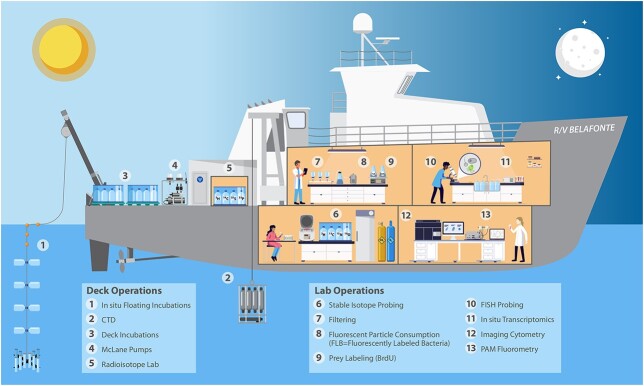
An idealized phago-mixotrophy focused research cruise: experiments and observations that use multiple tools and techniques for understanding phago-mixotrophy *in situ*. Coordinated efforts among researchers with varied expertise to combine classical methods (that quantify photosynthesis, prey ingestion and environmental parameters) alongside emerging methods can help (i) validate new methods, (ii) build consensus across the community on the appropriate approaches and (iii) target open questions about the evolution, traits and trade-offs, biogeography, and biogeochemistry of mixoplankton that can help inform and validate models. The image depicts typical shipboard equipment (1–4) and several methods listed in Table II (5–13), all of which can be used to study mixoplankton and phago-mixotrophy in some way. Illustration by Lee Ann Deleo (Skidaway) and Ashley Maloney.

To date, most of our current understanding of mixoplankton and phago-mixotrophy is based on cultured organisms within a laboratory setting. Studies have been conducted *in situ* that examine basic data on mixoplankton or the potential for phago-mixotrophy. For example, there has been an effort to assess mixoplankton presence or absence in the natural environment and estimate community ingestion rates (Domaizon *et al.*, 2003; Unrein *et al.*, 2007; Leles *et al.*, 2017; Faure *et al.*, 2019). However, there is not enough *in situ* data given the importance and ubiquitous nature of mixoplankton. Studies addressing more complex questions related to the role of mixoplankton in the natural environment are nearly absent. Currently, there is little consensus in the aquatic scientific community on the appropriate methods to study mixoplankton *in situ* or how various techniques can be optimized (but see Beisner *et al.*, 2019; Wilken *et al.*, 2019). A preliminary understanding of each research priority discussed above can begin with laboratory cultures, but the ultimate goal is to be able to address questions related to these topics within the natural mixoplankton community. This makes the development of *in situ* methods a necessary priority. Developing and testing new methods will require simultaneously applying multiple approaches, old and new, in order to compare results across all methods (Fig. 4).

Conducting *in situ* research on mixoplankton has been hindered by the limitations of current popular methods for detecting phago-mixotrophy within the natural environment. Differentiating mixoplankton from other planktonic constituents can be challenging; however, a variety of methods have been used to identify mixoplankton and estimate rates of phagotrophy and phototrophy (Table II; Beisner *et al.*, 2019; Wilken *et al.*, 2019; Mitra *et al.*, 2021). These methods include the use of fluorescently labeled particles (cellular prey or plastic microspheres) in combination with epifluorescence microscopy or flow cytometry to identify and sort *in situ* protists that ingest these particles. Additionally, the use of fluorescence *in situ* hybridization (FISH) or food vacuole staining using acidotropic probes (e.g. LysoTracker Green; Rose *et al.*, 2004; Anderson *et al.*, 2017) can be used to identify mixoplankton within the environment. More recent methods for exploring phagotrophy *in situ* use combinations of DNA stable isotope probing (Orsi *et al.*, 2018), RNA stable isotope probing (Frias-Lopez *et al.*, 2009), FISH (Massana *et al.*, 2009) or other prey labeling techniques (e.g. 5-Bromo-2’-Deoxyuridine (BrdU); Gast *et al.*, 2018). Although these methods estimate ingestion rates among *in situ* mixoplankton, there are methodological drawbacks (Wilken *et al.*, 2019) for each method that limit widespread use.

**Table II:** Summary of old and new methods used to study mixoplankton. Currently available methods for detecting and assessing phago-mixotrophy in culture and *in situ*. For each method we indicate whether it can be used to identify a mixoplankton cell, be used to taxonomically classify mixoplankton, estimate mixoplankton cell abundance, estimate the ingestion rate of mixoplankton, estimate the photosynthetic rate of mixoplankton, and whether it has ever been used to study *in situ* mixoplankton

Method	Is a cell mixoplankton?	Taxonomic identification	Abundance	Ingestion/Grazing rate	Photosynthetic rate	Has been used for *in situ* detection
Fluorescent particle consumption^1,2,3,5,7,8,9^	Yes	Some	Yes	Yes	No	Yes
Food vacuole staining^4,5,6,7,8^	Yes	Some	Yes	No	No	Yes
FISH probing^10,11,12,13^	Yes^a^	Yes^a^	Yes^a^	No	No	Yes^a^
Stable isotope probing^14,15,16^	Yes	No	Yes	No	Yes	Yes
BrdU labeling^17^	Yes	Yes	Yes^b^	No	No	Yes
*In situ* transcriptomics^18^	Yes	Yes	No	No	No	Yes
PAM fluorometry^19^	Yes	No	Yes	No	PE	Rare
Radioisotope probing^20,21,22^	Yes	No	No	Yes	Yes	Yes
Imaging cytometry^23^	Yes^a^	Yes^a^	Yes	No	No	Yes^a^

PE = photosynthetic efficiency

^a^Known mixoplankton only

^b^By OTU (operational taxonomic unit)

*References cited*: ^1^Cucci *et al.*, (1989), ^2^Havskum and Reimann, (1996), ^3^Unrein *et al.* (2007), ^4^Li *et al.*, (2016), ^5^Wilken *et al.* (2019), ^6^Rose *et al.* (2004), ^7^Anderson *et al.* (2017), ^8^Beisner *et al.* (2019), ^9^Bock *et al.* (2021), ^10^Unrein *et al.*, (2014), ^11^Hartmann et al., (2013), ^12^Grujcic et al., (2018), ^13^Massana *et al.* (2009), ^14^Terrado *et al.* (2017), ^15^Orsi *et al.* (2018), ^16^Carpenter *et al.* (2018), ^17^Gast *et al.* (2018), ^18^Lambert *et al.* (2022), ^19^Lin and Glibert (2019), ^20^Adolf *et al.* (2006), ^21^Zubkov and Tarran (2008), ^22^Duhamel *et al.* (2019), ^23^Brownlee *et al.* (2016)

Alongside established methods, emerging *in situ* techniques are being developed to provide further understanding of mixoplankton. Promising pathways include investigating patterns of gene expression associated with phagotrophy and photosynthesis through *in situ* metatranscriptomic analyses as discussed in the *Biogeography and Ecological Determinants* section. Furthermore*, in situ* single-cell techniques are under rapid development. These include a combination of imaging and stable isotope probing for the identification and quantification of both carbon and nutrient uptake rates of individual mixoplankton including photosynthesis–grazing ratios (Beisner *et al.*, 2019). For example, nano-scale secondary ion mass spectrometry (nanoSIMS or nanoSIP) whereby ^13^C- and/or ^15^N-labeled bacteria can be used to determine phagotrophy rates and ^13^C-labeled sodium bicarbonate can assess photosynthetic activity within specific protists (Terrado *et al.*, 2017; Carpenter *et al.*, 2018). Alternatively, cell sorting and other microfluidic techniques in association with Raman spectroscopy or Raman microscopy could assist with the identification, downstream cultivation or molecular characterization of isotope-labeled mixoplankton (Pinho and Hartman, 2017).

Flow cytometry in coordination with imaging capabilities can provide rapid identification and quantification of mixoplankton where there is *a priori* knowledge of trophic status based on previous literature. When images of protists exhibit fluorescence from acidotropic stains or fluorescently labeled tracers, in addition to chlorophyll fluorescence, morphotypes may be identified as potential mixoplankton. Flow cytometric imaging (i.e. Imaging FlowCytobot: McLane Research Laboratories and FlowCam: Fluid Imaging Technologies) combined with cell staining (Brownlee *et al.*, 2016) for long-term *in situ* deployments will allow for greater insight into seasonal and long-term dynamics of potential mixoplankton associated with environmental and climate change. Instrumentation such as the Amnis ImageStream^Ⓡ^ (Luminex), a multispectral imaging flow cytometer, combines flow cytometry with the fluorescence imaging of a microscope (George *et al.*, 2004). Along with brightfield images, fluorescence signatures can be visualized within the cell. While typically used to study morphology and metabolic activity (Dapena *et al.*, 2015; Hildebrand *et al.*, 2016), this instrumentation allows for rapid and high throughput *in situ* studies that use fluorescence to identify phago-mixotrophy. Furthermore, multiwavelength-excitation fluorometers have been used to distinguish the dynamics of predator and prey in mixoplankton co-cultures (e.g. Lin and Glibert, 2019).

In addition to detecting phago-mixotrophy and measuring ingestion and photosynthetic rates, tools are needed to quantify transfer of carbon, nutrients, trace metals and specific metabolites from the prey to the predator. To date, radioisotope tracers have helped estimate *in situ* bacterivory rates (Zubkov and Tarran, 2008; Hartmann *et al.*, 2012; Duhamel *et al.*, 2019;). Stable isotope addition methods have been applied to aquatic systems using isotopically labeled prey (Frias-Lopez *et al.*, 2009; Orsi *et al.*, 2018), organic and inorganic carbon (Schubotz *et al.*, 2015; Berthelot *et al.*, 2019, 2021), nutrients (Fawcett *et al.*, 2011), water (Ferrón *et al.*, 2016) and multiple labeled entities together (Wegener *et al.*, 2016; Terrado *et al.*, 2017). Targeting these tracer methods to mixoplankton can help track the incorporation of labeled atoms through phagotrophy and digestion to identify the fate of ingested material. Tracing isotope uptake (either stable or radiogenic tracers) back to mixoplankton *in situ* requires flow cytometric cell sorting (e.g. Zubkov and Tarran, 2008; Hartmann *et al.*, 2012; Duhamel *et al.*, 2019) and/or sequencing labeled nucleic acids (Wang and Yao, 2021) and taking advantage of group-specific biomarkers (Dijkman *et al.*, 2009; Close, 2019).

The potential to assess phago-mixotrophy in the past is invaluable for insight on our changing oceans and lakes. Lipid hydrogen isotope (^2^H/^1^H) ratios (Sessions *et al.*, 1999) and their response to metabolism in microbes (Zhang *et al.*, 2009; Heinzelmann *et al.*, 2015; Wijker *et al.*, 2019), plants (Gamarra and Kahmen, 2015; Cormier *et al.*, 2018, 2019) and to animal trophic ecology (Vander Zanden *et al.*, 2016), indicates that natural abundance stable isotope ratios of phytoplankton lipid biomarkers in the water column and sedimentary archives can provide important information about physiological shifts related to osmotrophy (Estep and Hoering, 1981; Cormier *et al.*, 2022). This could set the stage for future studies to target phagotrophic phytoplankton using hydrogen isotope ratios. The largest challenge in sedimentary analysis will be distinguishing between changes due to temperature, salinity, nutrients, light and phago-mixotrophy, which may all influence natural abundance hydrogen isotope ratios of biomarkers. Combining this approach with the emerging use of ancient DNA in sediments (Armbrecht, 2020) is a promising avenue for understanding past planktonic communities and may provide insight into unanswered questions raised in the *Evolution* section.

The major drawback to most emerging methods is the resources required to conduct the shipboard/field or laboratory experiments, followed by labor-intensive and expensive sample analysis. The ability to remotely detect active and inactive mixoplankton using sensors on sea-going autonomous instruments and satellites would be the ultimate contribution for characterizing large-scale seasonal to decadal variability. Overall, there is a need to assess the contribution of phago-mixotrophy with regard to distribution, biogeography and their contribution to biogeochemical models, so the continued development of *in situ* methods is key to understanding the roles these microbes play in a changing climate. For each research priority discussed, many new and improved methods are required to appropriately assess phago-mixotrophy within the natural environment.

## CONCLUSION

By distinguishing mixoplankton from zooplankton and phytoplankton, a new field of research with a wide range of possible questions to address in all aquatic environments has opened. Through outlining the general research priorities here, we hope to inspire a large number of scientists to contribute to this developing field. Ideally, in the coming years, several large-scale research projects will be underway that combine a diversity of expertise to address questions related to one (or more) of the outlined priorities. Even a single project where scientists from different research laboratories participate in the same research cruise—each addressing their question related to mixoplankton and phago-mixotrophy within the same region—would be a major leap forward in our current understanding of this important functional group.

## Data Availability

There are no data associated with this article.
